# Chronic Pelvic Pain: Assessment, Evaluation, and Objectivation

**DOI:** 10.1155/2017/9472925

**Published:** 2017-11-20

**Authors:** Maria Beatrice Passavanti, Vincenzo Pota, Pasquale Sansone, Caterina Aurilio, Lorenzo De Nardis, Maria Caterina Pace

**Affiliations:** Department of Woman, Child, General and Specialized Surgery, University of Campania L. Vanvitelli, Naples, Italy

## Abstract

Chronic Pelvic Pain (CPP) and Chronic Pelvic Pain Syndrome (CPPS) have a significant impact on men and women of reproductive and nonreproductive age, with a considerable burden on overall quality of life (QoL) and on psychological, functional, and behavioural status. Moreover, diagnostic and therapeutic difficulties are remarkable features in many patients. Therefore evaluation, assessment and objectivation tools are often necessary to properly address each patient and consequently his/her clinical needs. Here we review the different tools for pain assessment, evaluation, and objectivation; specific features regarding CPP/CPPS will be highlighted. Also, recent findings disclosed with neuroimaging investigations will be reviewed as they provide new insights into CPP/CPPS pathophysiology and may serve as a tool for CPP assessment and objectivation.

## 1. Introduction

Pain is an unpleasant sensory and emotional experience associated with actual or potential tissue damage or described in terms of such damage [1]. Chronic Pelvic Pain (CPP) is defined by the European Association of Urology (EAU) as “chronic or persistent pain perceived in structures related to the pelvis of either men or women. It is often associated with negative cognitive, behavioural, sexual and emotional consequences as well as with symptoms suggestive of lower urinary tract, sexual, bowel, pelvic floor or gynaecological dysfunction. In the case of documented nociceptive pain that becomes chronic/persistent through time, pain must have been continuous or recurrent for at least 6 months. If non-acute and central sensitization pain mechanisms are well documented, then the pain may be regarded as chronic, irrespective of the time period” [2]. The American College of Obstetricians and Gynaecologists similarly defines CPP as “non-cyclic pain lasting for 6 or more months, that localizes to the anatomic pelvis, anterior abdominal wall at or below the umbilicus, the lumbosacral back, or the buttocks and is of sufficient severity to cause functional disability or lead to medical care” [3]. The EAU further defines Chronic Pelvic Pain Syndrome (CPPS) as “the occurrence of CPP when there is no proven infection or other obvious local pathology that may account for the pain. It is often associated with negative cognitive, behavioural, sexual or emotional consequences, as well as with symptoms suggestive of lower urinary tract, sexual, bowel or gynaecological dysfunction. CPPS is a subdivision of CPP. […] Pain perception in CPPS may be focused within a single organ, more than one pelvic organ and even associated with systemic symptoms such as Chronic Fatigue Syndrome (CFS), Fibromyalgia (FM) or Sjögren's Syndrome. When the pain is localized to a single organ, some specialists may wish to consider using an end organ term such as Bladder Pain Syndrome (BPS). The use of such a phrase with the terminology ‘syndrome' indicates that, although peripheral mechanisms may exist, CNS neuromodulation may be more important and systemic associations may occur. When the pain is localized to more than one organ site, the term CPPS should be used.” [2]. “End organ” terminology reflects the site in which pain presents, and therefore specific terms for the involved organ (such as Bladder Pain Syndrome and Prostate Pain Syndrome) are classified in EAU guidelines. In more detail, the guidelines distinguish Urological, Gynaecological, Gastrointestinal, and Musculoskeletal Pain Syndromes [2]. Urological Pain Syndromes include Bladder Pain Syndrome, which is often termed as “Interstitial Cystitis” by several authors, and Prostate Pain Syndrome, which is often termed “Chronic Prostatitis/Chronic Pelvic Pain Syndrome” instead by many authors according to the NIH Classification of Chronic Prostatitis [4]. Moreover, Scrotal Pain Syndrome, Testicular Pain Syndrome, Epididymal Pain Syndrome, Postvasectomy Scrotal Pain Syndrome, Penile Pain Syndrome, and Urethral Pain Syndrome are all classified as Urological Pain Syndromes. Again, in many articles authors refer to these syndromes as “Chronic Orchialgia” or “Chronic Scrotal Syndrome” [5, 6]. Gynaecological Pain Syndromes include Vulvar Pain Syndrome (also termed “Vulvodynia”), Vestibular Pain Syndrome, Clitoral Pain Syndrome, Dysmenorrhea, and Endometriosis-Associated Pain Syndrome. Irritable Bowel Syndrome and Pelvic Floor Muscle Pain Syndrome are the most widespread Gastrointestinal and Musculoskeletal Pain Syndromes, respectively.

Notably, EAU guidelines underscore the fact that central sensitization mechanisms and Central Nervous System (CNS) neuromodulation and neuroplasticity are relevant pathophysiological mechanisms in CPP/CPPS, and indeed many patients (up to 50% in some cohorts) report neuropathic pain symptoms [7–10]. In particular, Endometriosis appears to be deeply connected to sensitization and neuroplasticity mechanisms [9, 11], although neuropathic pain features may occur in virtually any CPP/CPPS patient [12, 13]. Specific questionnaires for neuropathic pain assessment will be discussed later in this review, and dedicated laboratory tools to assess neuropathic features will be discussed as well. Moreover, since central neuroplasticity and neuromodulation are relevant features of CPP, the role of functional brain neuroimaging in CPP/CPPS patients will also be discussed in a dedicated section.

While acute pelvic pain is considered as the fifth vital sign [14, 15] in the same way as other acute pain conditions, CPP is generally considered to be a description of a clinical condition [16] rather than a diagnosis. Nevertheless, CPP has a significant impact on women of reproductive and nonreproductive age, with a considerable burden on overall quality of life (QoL) and on psychological, functional, and behavioural status. CPP prevalence varies in a wide range, according to different cohort sampling, from 5,6% to 30,9% [17–20]. Notably, the reported prevalence varies according to the country in which the sample is enrolled: in a recent epidemiology-based study prevalence ranged from 6,4% in Mexico to 25,4% in New Zealand [21]. This wide range of reported prevalence may reflect sociocultural differences in the investigated countries with a possible resilience to the admission of urogynaecological troubles. The prevalence of CPP/CPPS in men is also variable in different studies, although lower than their prevalence in women; the reported prevalence of CPP symptoms in men ranges between 2% and 17% [19, 22–24]. Prostate Pain Syndrome/Chronic Prostatitis and Scrotal Pain/Orchialgia are the most common syndromes male patients complain of. Prostate Pain Syndrome/Chronic Prostatitis is a high prevalent syndrome (prevalence ranges between 4,5 and 9% in different cohorts, although the effective prevalence is likely underestimated) [25], which is often accompanied by psychosocial burdening symptoms, Voiding Cycle Lower Urinary Trait Symptoms and Bladder Pain [26], Postejaculatory Pain [27], Erectile Dysfunction [28], and Pelvic Floor Muscle Pathology [29]. In a recent survey regarding young Canadian and African males with age ranging from 16 to 19 years [30, 31], Chronic Prostatitis-like symptoms were assessed, and also sexual, psychological, and overall QoL features were investigated; 8–13,3% of mild symptoms and 3–5,4% of moderate to severe symptoms were identified in the two cohorts, respectively. In both the cohorts, pain, urinary, and psychological symptoms were predictive of a worsened QoL.

Chronic Scrotal Pain is as well a likely underestimated syndrome, with reported prevalence ranging between 2,5 and 5% [5, 6, 32, 33]. It is as well accompanied by poor psychosocial, sexual, and QoL outcomes and thus represents a considerable burden for patients.

Pelvic Floor Muscle Pathology and widespread muscle tenderness deserve particular mention; they are recurrent features both in men and women affected by CPP/CPPS, as well being internal and external Pelvic Tender Points [34–38]. Objectivation tools for these relevant features of CPP/CPPS patients such as Quantitative Sensory Testing and Algometry will be further discussed in a dedicated section.

Although epidemiologic data vary in this great magnitude, CPP represents a significant socioeconomic problem, since Pelvic Pain Syndromes do have an impact on QoL and often result in depression, anxiety, impaired emotional functioning, and fatigue [39–44]. Sexual behaviour and intercourses are often impaired and disturbed by painful syndromes both in men and women. Some patients, both male and female, can even present pain catastrophizing features, which are exaggerated negative responses to imagined pain or actual pain, affecting an individual's belief system and coping strategies [45]. Pain catastrophizing has been recognized as an essential risk factor for chronic pain and could also serve as an important predictor of cognitive distress, pain-related disability, analgesic use, and dysfunctional adjustment to pain in clinical situations [46–50].

Pelvic pain affects women more frequently than men because of genetic, hormonal, sociocultural, environmental, and anthropological aspects. From an evolutionary perspective, men have always been “warriors and hunters” and consequently they more often experienced somatic pain, which is qualitatively and quantitatively different from visceral pain. Otherwise, visceral pain has always been reported mostly by women because of menstrual-related pain and painful delivery experiences.

From a biological and pathophysiological point of view, women complain of pelvic pain more than men because of both qualitative and quantitative differences in pain modulation pathways. In fact, women are more sensitive to opioid *κ* receptor mediated analgesia than men (qualitative aspect). Furthermore, they are less sensitive to pain modulation, and female pain threshold is lower than that of men (quantitative aspect) [15, 16, 51]. The reason for such differences probably depends on sex hormones levels with related pharmacodynamic and pharmacokinetic (opioids absorption, distribution, and metabolism) implications. Several studies reported an estrogen-related modulation of cerebral opioid peptides levels, cerebral opioids mRNA concentration, and opioid receptor density and signalling [16, 51, 52]. Furthermore, estrogens can attenuate endogenous and exogenous opioids effects through a direct link to opioid receptors [52] and can interfere with long term pain modifications in both Central and Peripheral Nervous System. As inflammatory and neuromodulating mediators received growing attention in recent years [53] in order to disclose acute (inflammatory) and chronic pain mechanisms, several researches have been assessed sex-related differences in the modulation of pain pathways. Recent investigations showed that female sex hormones, in particular 17*β*-estradiol and progesterone, are able to influence the levels of PGE2 and CGRP in the Periaqueductal Gray (PAG) [54]; moreover, a lower level of 17*β*-estradiol and/or progesterone augments PGE2 and its EP3 receptor, and PGE2 plays a role in regulating the expression of CGRP in the PAG. Different levels of 17*β*-estradiol were also able to influence the release of CGRP from murine F11 cells [55]. Moreover, a relationship between 17*β*-estradiol and pain mediators was documented in a female mice model of temporomandibular Joint arthritis [56]. It was found that 17*β*-estradiol upregulates both NGF and TRPV1 in a dose-dependent manner, and, in particular, synovial TRPV1 is involved in allodynia of temporomandibular joints and was responsible for COX-2 overexpression in inflamed joints. Such results seem to be relevant both for pathophysiological mechanisms of pain and also for the future development of new therapeutic pharmacological targets. Testosterone, on the other hand, is also known to be relevant in both behavioural development and pain modulation mechanisms, as it has been demonstrated in murine models that opioid analgesic sensitivity is influenced by testosterone levels even in early life in parallel with sexual differentiation of the reproductive system [57]. Other more recent researches assessed sex differences in analgesic response to antiandrogen and antiestrogen drugs in mice models of urethral calculus [58], and it was also underscored that estrogens and testosterone can interfere differently with visceral pain-related behavioural response in female and male rats [59], thus confirming that sex hormones in the two genders can act differently in enhancing/inhibiting pain perception and modulation. Pregnancy and delivery also appear to be relevant triggers to pain pathways modifications: animal studies performed in mice suggest an increased pain threshold in pregnancy, in particular immediately before delivery. Spinal opioid *κ* and *δ* receptors and the pain modulating descending noradrenergic pathways seem to be implicated [51]. Moreover, the same studies demonstrate sex-related specificity of neural pathways in the spinal cord, in the Periaqueductal Gray (PAG), and in the rostral ventromedial medulla. Sex differences in pain perception and pain modulation may thus have been maintained throughout evolution in mammalians, as sex hormones influence on neural pathways, menstruations, pregnancy, and delivery is shared among species and extends, although slightly differently, also to humans [60–62]. Despite the recent advances in research, further investigations are necessarily warranted to better evaluate both physiological and pathological mechanisms of sex-related differences in pain perception/modulation, as better understanding of this intricated arras may lead to better tailored clinical management and therapeutic strategies for male and female CPP/CPPS patients.

As mentioned earlier, CPP and CPPS are a major burden for patients and moreover imply diagnostic and therapeutic difficulties in many cases: that is, up to 55% of women with CPP/CPPS have no clear and definite pathological findings even after laparoscopic evaluation [10, 63]. Notably, pain intensity and quality reflect pathophysiological mechanisms underlying the symptoms, and therefore evaluation, assessment, and objectivation tools are often necessary to properly address each patient and consequently his/her clinical needs. Nevertheless, objectivation of pain is often a troublesome task, as pain is a subjective experience and different patients complain of it in several ways according to age, gender, and education beyond pain intensity and qualities themselves. Pain evaluation key points are patient's anamnestic interview and clinical examination, pain intensity and characteristics evaluation, patient psychological evaluation, and finally the planning of a therapeutic protocol with daily evaluation of the effects. Interview and clinical examination are still unneglectable tools for pain evaluation, as they allow the physician to know the characteristics of the painful experience of the patient and in some cases the distinction between different pain features. Nevertheless, in many times a clinical assessment based only on the examination may not yield a satisfactory diagnosis even when experienced physicians are involved, and diagnostic tools for a better assessment of pain characteristics and a complete evaluation of patient's symptoms are often warranted. A suitable qualitative and quantitative pain analysis allows, indeed, a faster and more complete diagnosis. The usefulness of these methods is of remarkable importance for CPP especially in case of difficult diagnosis and less experienced physicians [64]. Pelvic pain measurement includes both verbal and instrumental methods to choose from according to pain duration (acute or chronic), pain pathophysiology, gender, age, and education. Instrumental methods allow better objectivation of pain and may be useful to document an unspecific and subjective symptom such as pain, thus converting it into a well demonstrable sign. Here we review the different tools for pain assessment, evaluation, and objectivation; specific features regarding CPP/CPPS will be highlighted in each section.

*Take-Home Message*. Many patients, both male and female, suffer from CPP/CPPS during their life span. CPP/CPPS is accompanied by psychosocial and mood disturbances, worsened sexual functioning, decreased overall QoL, and occasionally even catastrophizing features. Despite this, CPP and CPPS are subtle, underestimated, and underdiagnosed clinical conditions; also, as different features are often present in the same patient (i.e., pain, muscle tenderness, urinary tract symptoms), correct and satisfied management of patients is seldom feasible. Moreover, as in other pain syndromes, objectivation and evaluation of pain are often troublesome both for patients and physicians.

## 2. CPP/CPPS Assessment and Objectivation Tools

### 2.1. Pain Scales and Questionnaires

Besides anamnestic interview and clinical examinations, pain scales and questionnaires are useful and reliable tools to assess pain intensity, and moreover some questionnaires also allow the distinction between nociceptive and neuropathic pain in patients presenting with more complex painful syndromes. Notably, several scales and questionnaires have been developed over years and the choice for the proper one depends on clinical settings; patient's gender, age, and education; and physician's personal experience. We also will review some QoL and psychological assessment questionnaires, as CPP and CPPS often have a detrimental impact on patients' daily activity and represent a considerable burden on emotional and psychological processing. Moreover, as discussed above, patients presenting with associated psychological features besides CPP/CPPS often have poorer pain control and worse clinical course than patients without these features. Recently, some criteria for the reevaluation of scales and questionnaires in chronic pain patients have been proposed [65]. The proposed criteria are content validity (scales should really measure the most important symptoms associated with pathology), test–retest reliability (a patient should give the same answer concerning the same type of pain in case of repeated administration of the same test), and responsiveness (scales results and patient conditions should change in the same way). Below we will review the most widespread scales and questionnaires that might be useful for CPP/CPPS assessment and evaluation.

#### 2.1.1. Monodimensional Pain Scales

Monodimensional pain scales are a simple yet useful tool for a “raw” first assessment of pain. As they merely consist of a value of patient's experienced pain, they are not suitable to reveal the quality of the painful experience or to differentiate nociceptive from neuropathic features of pain. Nevertheless, they are still useful for both acute [15] and chronic pain evaluation, as they are quickly administrable in inpatient and/or outpatient service. The most widespread monodimensional pain scales are the Visual Analogical Scale (VAS) and the Numerical Rating Scale (NRS), which assess pain with an increasing number from 0 (no pain) to 10 (the worst pain imaginable). Another commonly used scale is the Verbal Numerical Rating Scale (VNRS), in which the patient is asked to match pain with an adjective (and a corresponding number). Since they consist in numeric values, monodimensional pain scales are still often used as an outcome measure for clinical trials [66], both for measuring treatment effectiveness [67] and for validating new developed diagnostic tools [68]. All the three scales are comparable in pain evaluation, although different authors may prefer one to the others [69–72]. Wong–Baker Faces Pain Rating Scale is instead a scale which is suitable for speechless patients, as they are asked to indicate a drawn face that is representative of the pain; the drawings are then matched again with corresponding numbers. Lastly, a pain intensity score may also be obtained with a behavioural index, which is the esteem of pain evinced from patient's behaviour. A specific behavioural index, which also included psychological and neurovegetative signs, has been designed for gynaecologic pain evaluation in emergency settings [15].

#### 2.1.2. Multidimensional Pain Scales

These questionnaires allow a more complex pain evaluation beyond asking the patient to merely evaluate the intensity of the painful experience; other relevant features are investigated. The nature and location of pain are assessed, along with the esteem of the impact of pain on daily life activities and mood. Multidimensional pain scales are therefore a useful tool for chronic pain evaluation.

Mc Gill Pain Questionnaire (MPQ) is a widespread multidimensional pain questionnaire. MPQ assesses the intensity of the present pain with a 0–5 scale; moreover, it allows examining pain distribution with a picture representing the human body on ventral and dorsal planes suitable for pain distribution mapping. Further, pain qualities and psychological processing are described by the patient by answering a series of specific questions divided into clusters (sensory discriminative, cognitive evaluative, and motivational affective) [73]. As MPQ, despite being a reliable and useful tool for chronic pain, is very time consuming, a short form of Mc Gill Pain Questionnaire (SF-MPQ), with strong and significant correlation with MPQ, has been developed [74]. It is an easier and more rapidly administrable version of MPQ, consisting of 15 descriptors (11 sensory, 4 affective) which are rated on an intensity scale from 0 to 3. Both the questionnaires have been translated into several languages to make them more suitable in different sociocultural environments. The Brief Pain Inventory (BPI) [75] is another multidimensional questionnaire which assesses the severity of pain and its interference with patients' daily activities. Pain localization, drugs intake, and pain relief are also evaluated. This test could be carried out as a self-report, an interview, or an interactive vocal response system (IVR). The Brief Pain Inventory (BPI) is available in two formats: the BPI short form, which is used for clinical trials and is the version used for foreign-language translations, and the BPI long form, which contains additional descriptive items that may be clinically useful (descriptors of pain, such as “burning” or “tingling”).

#### 2.1.3. Neuropathic Pain Assessment Questionnaires

In order to distinguish nociceptive from neuropathic pain features, properly tailored, adequate therapy and screening tools for neuropathic pain are also useful in CPP/CPPS patients; besides the interviews, some tools also include physical tests and examinations. The Neuropathic Pain Scale [76] examines dysesthetic features of pain; it is administrable in an average time of 10 minutes but may be restricted by the level of education of the patient (if lower) and in the experience of the examiner (if limited). PainDETECT [77] is a self-report questionnaire which includes a schematic drawing of pain distribution and esteem of pain fluctuations in time. It, therefore, allows the patient to describe the timing, localization, and radiation of pain, along with any other pain characteristics. PainDETECT is a reliable screening tool with high (around 80%) sensitivity and specificity. The Leeds assessment of neuropathic symptoms and signs (LANSS Pain Scale) [78] is another questionnaire that allows the differentiation between nociceptive and neuropathic pain and the evaluation of the qualitative characteristics of the neuropathic pain itself; compared to clinical diagnosis, its sensitivity and specificity are 82–91 and 80–94%. ID Pain [79] can distinguish nociceptive and neuropathic pain features, with satisfactory accuracy. It is a six-item questionnaire, and for each item the patient is asked to give an affirmative or negative answer and a numeric value from −1 to 5. The total score, resulting from the sum of the scores for each item, will allow discriminating the type of pain. Neuropathic Pain Symptoms Inventory (NPSI) [80] is a questionnaire composed of 12 items (for spontaneous ongoing pain, paroxysmal pain, evoked pain, and paresthesia/dysesthesia). Each of these items is quantified on a 0–10 numerical scale. The discrimination and quantification of five distinct clinically relevant dimensions of neuropathic pain syndromes and their sensitivity to treatment are also assessed. New Neuropathic Pain Diagnostic Questionnaire (DN4) [81] is very easy to administer (only 4 questions) and to evaluate (patient is asked to generate a positive/negative sentence). If the score is equal to 4 or higher, the pain is likely to be neuropathic. The comparison between the diagnostic accuracy of the LANSS Pain Scale and the DN4 for the detection of peripheral neuropathic pain showed that, although both questionnaires are good screening tools, the DN4 questionnaire is particularly recommended for identifying patients with neuropathic pain in clinical practice and research studies [82]. Neuropathic Pain Questionnaire [83] includes 12 different items which help the physician to distinguish neuropathic from nonneuropathic pain. The questionnaire allows assessing negative sensory symptoms (i.e., numbness), positive sensory symptoms such as paresthesia and dysesthesia (often referred to as “tingling pain”), and eventual increase of pain after tactile stimulation. It does not include physical examination measures and is, therefore, not highly recommended.

#### 2.1.4. CPP/CPPS Specific Questionnaires

Specific questionnaires for assessment and evaluation of CPP/CPPS have also been developed, both for diagnostic and therapeutic purposes. Pelvic Pain and Urgency/Frequency Questionnaire (PUF) is a self-report questionnaire designed to investigate the presence of urinary urgency/frequency, pelvic pain, or sexual dysfunction in both sexes and specifically developed by C. Lowell Parsons for the study and diagnosis of Interstitial Cystitis (IC/PBS: Interstitial Cystitis/Painful Bladder Syndrome). It examines both a symptom score (which measures how often a patient experiences problems) and a bother score (how much symptoms bother the patient); combination of both scores is the total PUF score. Score range is between 0 and 35 and a score equal to or greater than 12 is suggestive for the disease in question even if further diagnostic urological, gynaecological, or urogynaecological evaluations are needed. It was, in fact, demonstrated that high scores may be compatible with different diagnoses such as urinary tract infections or overactive bladder syndrome [84]. To date, however, the PUF questionnaire is mainly used in the followup of patients already being treated for urological disorders. Several other scales and questionnaires have been developed in order to assess and evaluate Interstitial Cystitis/Painful Bladder Syndrome patients. One of the most widespread is the O'Leary-Sant Interstitial Cystitis Symptom Index and Problem Index [85] which consist in two self-administrable indices that measure pain and urinary symptoms and their impact (the problem index) on overall QoL and daily activities. Psychometric performance of both the indices was reported to be good, with the symptom index being able to distinguish between patients' and controls' characteristics [86]. The indices also showed good responsiveness [87]. Both indices should be useful in the evaluation and management of patients with IC/PBS; accordingly, they were able to predict treatment outcomes in clinical trials [88]. Similarly, the Bladder Pain/Interstitial Cystitis Symptom score, developed by Humphrey and colleagues [89], was validated in order to properly evaluate IC/PBS patients and correctly allocate them in clinical trials. The examined items are focused on bladder and urinary issues: bladder pain, persistent urge to urinate, and high urinary frequency. The University of Wisconsin Interstitial Cystitis Scale [90] is a symptom scale originally developed for women with Interstitial Cystitis. The scale contains 7 questions directly related to bladder symptoms (bladder pain, bladder discomfort, burning bladder sensation, nycturia, sleep difficulties due to nycturia, polyuria, and urinary urgency) in the last 24 hours. The Pelvic Pain Assessment Form is another gender-specific scale designed for women and developed by the International Pelvic Pain Society [91] and is composed of two parts: the first should be filled out by the patient and the last by the physician. The form allows complete registration of information about the patient's pain (causes, duration of pain, Visual Analogue Scale score, pain medications, and pain distribution maps), patient's past and recent clinical history (including surgical, obstetrical, family, medical, menstrual, and gastrointestinal history; eating and health habits; genitourinary symptoms; coping mechanisms; sexual and physical abuse history), and eventual former evaluations performed by other physicians. The form also includes a short form of Mc Gill Pain Questionnaire and a section dedicated to a pelvic varicosity pain syndrome specific questionnaire. The physician will also provide a physical examination, a gynaecological examination, and lastly a diagnostic and therapeutic plan. Notably, this form does not have a diagnostic value. The National Institute of Health Chronic Prostatitis Symptom Index (NIH-CPSI) Questionnaire [92] is a 13-item index developed to assess symptoms and quality of life in men with Chronic Prostatitis/Chronic Pelvic Pain Syndrome (CP/CPPS) [93]. It has demonstrated good reliability, validity, and responsiveness to change [94], and it has been used as the primary outcome variable in multiple large-scale studies of CP/CPPS treatments. It has also been translated into multiple languages for international use [95, 96]. It is indicated for patients complaining of CPP, probably caused by Chronic Prostatitis or CPPS. Pain, voiding symptoms, and any interference of the symptoms with the quality of life are evaluated. The Genitourinary Pain Index (GUPI) is a more recent symptom index developed from NIH-CPSI [97] in order to assess genitourinary pain in both men and women. The GUPI included items dealing with urinary symptoms, localization and intensity of pain symptoms, and overall quality of life. Total scores range from 0 to 45; individual scores for the domains of pain, urinary symptoms, and quality of life impact can also be derived. Similarly, another questionnaire, the CPPQ-Mohedo, has been developed by Díaz Mohedo and colleagues [98]. It is also derived from NIH-CPSI score for male patients, with the purpose of editing a feasible questionnaire for both male and female patients with the same analysed items.

Recently, a novel phenotyping system for urological CPPS has been developed, the UPOINT system [99]. The acronym UPOINT stands for urinary, psychosocial, organ-specific, infection, neurological/systemic, and tenderness. It has been developed to stratify Interstitial Cystitis/Bladder Pain Syndrome and Chronic Prostatitis/Prostate Pain Syndrome according to six domains that allow patients' better assessment and evaluation, clustering and analysing the parts (i.e., the domains) of the acronym [100–102]. The UPOINT system is feasible for both genders and allows reliable and accurate match with severity of symptoms in each domain. Furthermore, several researches based patients' selection and treatment outcome measurement on UPOINT system scores [103], as the multidomain allocation of any symptom experienced by a patient allows a consequent multitargeted therapeutic strategy that is specially tailored for that patient and actually improves the various therapeutic outcomes in different trials [104–107].

Finally, a dedicated questionnaire has also been proposed for Vulvodynia patients [108], which might be helpful in assessing patients' symptoms and therefore recommending an adequate therapy.

#### 2.1.5. QoL and Psychological Assessment

CPP and CPPS are, according to their definition, usually associated with detrimental effects on overall QoL and with psychological comorbidities. Moreover, it has been shown that psychological impairment per se is responsible for worsened symptoms and is a predictor for bad therapeutic outcome (see Section 1). Therefore, for an exhaustive evaluation of CPP/CPPS patients, both QoL and psychological status should be assessed.

Several questionnaires explore the impact of pain symptoms on QoL in order to provide more complete management of the Chronic Pelvic Pain. Quality of life is best defined as patients perception of and reaction to their health status and nonmedical aspects of their lives (i.e., physical, functional, emotional, mental, and social wellbeing). QoL is measured with a collection of items, scales, domains, and instruments. In particular, instruments are classified as generic (i.e., SF- 36), disease-specific, site- or region-specific, dimension-specific (i.e., Mc Gill Pain Questionnaire), or utility measures (i.e., EuroQoL EQ-5D). To ensure an appropriate evaluation, the use of a disease-specific instrument in combination with a generic instrument should be recommended [109]. Short Form Health Survey (SF-36) evaluates different items of quality of life in various areas through 36 questions and is extremely reproducible and reliable. It is the most commonly used questionnaire for pelvic pain assessment [109]. There is also a shrunk version of SF-36, SF-12, which only examines 12 questions. EuroQoL (EQ-5D) is another reliable questionnaire [110], not disease specific, which meets internationally accepted standards. This simple and short questionnaire allows measuring the health status of the patients and their quality of life in order to evaluate overall health status. It consists of two distinct sections: The first is composed of five items (mobility, self-care, usual activities, pain/discomfort, and anxiety/depression) that affect the actual health status of the patient. Each item provides the possibility to choose between three (or more, recently five) different levels of severity. The second section of the questionnaire includes the EQ-VAS, thus allowing also pain assessment. The Pain Disability Index [111] is a psychometric assessment tool designed to help patients to measure the impact of chronic pain on daily activities. The patient is shown a form composed of various daily activities grouped into seven categories and is asked to indicate (through a 0 to 10 score) the perceived impairment. The PIQ-6 Pain Impact Questionnaire Package [112] is another tool which analyses the interference of pain with physical, mental, and social features. Pain Disability Questionnaire focuses on pain localization and radiation; time of the first episode of pain; previous events; pain description through adjectives; interference of pain with work, school, social life, family, sport, and or any other daily activity; previous therapies or medical examinations; efficacy of medication intake. Sexual functioning self-assessment is also available for both male and female patients [113, 114]; it might be worth evaluating this feature as well, as sexual function abnormalities are relatively frequent in CPP/CPPS patients of both genders.

Regarding psychological status, anxiety, depression, and catastrophizing may be assessed with proper questionnaires as well [115]. Patients with CPP/CPPS have an increased risk of developing psychological-psychiatric disorders even if the latter may preexist CPP/CPPS. We suggest that easy tools for psychological screening such as the Behaviour Illness Questionnaire, the Hamilton Psychiatric Rating Scale for Depression, and the Hospital Anxiety and depression Scale (HADS) should be used in order to provide a more complete and careful evaluation for CPP/CPPS patients. Also, catastrophizing may be assessed with a dedicate questionnaire [116].

*Take-Home Message*. Pain scales and questionnaires are fundamental tools when approaching, assessing, and evaluating CPP/CPPS patients. Mono- and Multidimensional scales are useful tools for patient assessment, for trials enrolment, and for therapeutic outcomes. CPP/CPPS specific questionnaires and indices are as well unneglectable tool for CPP/CPPS evaluation, as assessment and therapeutic interventions are better guided by scoring systems such as the UPOINT system. Finally, psychosocial and QoL scales as well should be borne in mind, as several patients report detrimental effects of CPP/CPPS on psychosocial functioning and overall QoL.

### 2.2. Pain Objectivation: Quantitative Sensory Testing and Electrophysiology

As mentioned above (see Section 1), neuropathic pain features are commonly found in many CPP/CPPS patients. Recognizing such cluster of patients has remarkable diagnostic and therapeutic value, as a proper therapy may be prescribed to those patients that experience dysesthetic/neuropathic symptoms. The objectivation of neuropathic pain features, although screening tools like questionnaires are helpful, is still a challenge for physicians. Quantitative Sensory Testing (QST) is a tool that measures the perception of mechanical, thermal, and painful stimuli delivered to determine the perceptive threshold for each sensation. Stimuli are delivered at increasing and decreasing intensities. Specific devices (i.e., von Frey hairs, weighted needles, vibrometers, and thermodes) are used to assess each sensory threshold. Several studies assessed QST changes in CPP/CPPS patients. A recent investigation comparing Neuropathic Pain Questionnaires and QST findings demonstrated [10] that only a few female CPP patients in the examined cohort have no changes in pain thresholds. Also, a positive correlation was found when matching QST results with questionnaires. QST assessment has been performed in several cohorts of CPP/CPPS patients with different techniques used to deliver the painful stimulation. Another recent investigation compared bladder sensitivity between patients with pelvic pain and patients who were pain-free by noninvasive and controlled bladder distension [37]. Reproductive-age women with nonbladder CPP, those with Painful Bladder Syndrome (PBS), and those who are healthy controls were enrolled. Participants were compared on cystometric capacity, pelvic floor PPTs, and pelvic muscle function. Participants with PBS exhibited higher bladder distension pain than those with CPP, with both groups reporting higher pain levels than controls. No significant associations were found between bladder distension pain and pelvic muscle structure or pain sensitivity measures; however, bladder distension pain positively correlated with both vaginal PPTs adjacent to the bladder and pain with transvaginal bladder palpation. Similar findings were also found in a previous study comparing CPP/PBS patients to healthy controls [117]: women with CPP or PBS exhibited enhanced pain sensitivity with lower PPTs than pain-free participants; also, prolonged pain aftersensation was documented in CPP/PBS patients. PPTs were also evaluated in male CPP patients affected by Urological Chronic Pelvic Pain Syndrome [118]; PPTs on 10 genitopelvic sites and one control site (deltoid) were measured, and UCPPS men had reliably lower pain thresholds compared to controls in all locations, including the deltoid. UCPPS men also demonstrated consistently lower overall pain thresholds regardless of location. Besides assessing a reduced PPT in pelvic area, this study also underscored widespread alteration in pain perception, shedding light on possible central mechanisms of pain perception in CPP/CPPS patients. This study assessed PPTs with a specific algometer, which is a dedicated device that administers a determined pressure to patients in order to evaluate pain thresholds. QST evaluation has also been performed in a cohort of men suffering from Erectile Dysfunction [119], which may often complicate the clinical history of men suffering from CPPS as discussed in Section 1; vibration, pressure, spatial perception, and warm and cold thermal thresholds were evaluated in 107 patients. It has been found that warm thermal thresholds were altered in Erectile Dysfunction patients versus controls, thus demonstrating a neurophysiological alteration in these patients.

Algometers have previously been used for the definition of the Myofascial Pain Syndrome (which may be responsible for CPP) [120] with the registration of PPTs, despite the lack of a gold standard in the definition of such syndrome. The study confirmed the usefulness of the pressure algometer for a more correct definition of Myofascial Pain Syndrome with the stimulation of the muscles of the anterior abdominal wall. In order to measure genital PPTs in CPP/CPPS patients, a specific algometer has been developed. It was suggested that pain threshold values measured through an internal (transvaginal) algometer can be considered a reliable and valid measures of pain sensitivity of the pelvic muscles compared with NRS values [121, 122]. Pain threshold values could be useful in the formulation of the diagnostic criteria for any disease associated with somatic and/or visceral pelvic pain. The authors found an association between pain threshold and the applied pressure value bringing on pain. The recordings were performed in 10 specific points: pubococcygeus, iliococcygeus, coccygeus, and obturator for each side as well as the anterior and posterior vaginal wall. The structure of a vaginal algometer includes a polyethylene terephthalate glycol housing, resembling a thin thimble, and fits on the distal tip of the examiner's finger as a thin shell. This fits underneath an examination glove and anatomical design minimizes any additional discomfort resulting from a normal musculoskeletal examination of the pelvis. The signal, originating from the palpation, is amplified and passes through a digital converter. A recent research investigated the relationship between nongenital tender point tenderness and intravaginal muscle pain intensity [123] in Vulvodynia patients with or without fibromyalgia. A significant correlation was found between numeric rating scale pain scores on the nongenital tender point tenderness exam and algometer testing on the iliococcygeus region and the posterior vaginal wall. Subjects with fibromyalgia by tender point tenderness had significantly higher iliococcygeal pain and posterior vaginal wall pain than women without fibromyalgia. Further, in order to measure external genitals PPT, a dedicated Vulvalgesiometer was also developed [124]. The authors performed a trial investigating different outcomes. First of all, they established that the Vulvalgesiometer was able to distinguish between women affected by Provoked Vestibulodynia (PVD) and healthy controls. Indeed, when comparing external genitals PPTs of the two cohorts, women affected by PVD showed significantly lower vestibular PPTs than unaffected women. Another assessed outcome was interrater reliability, which was assessed by PPTs measurement performed by two different investigators. As the Vulvalgesiometer also showed good interrater reliability, the authors concluded that it might serve as a tool for both clinical and research purposes. A more recent investigation evaluated PPTs in two groups of PVD patients, in order to assess differences between primary (PVD1) and secondary (PVD2) PVD patients by eliciting PPTs with a Vulvalgesiometer [125]. Also, questionnaires, psychophysical testing, and neuroimaging (both structural and functional) were examined as outcomes in order to disclose intergroup differences. Women with PVD1 showed lower PPTs compared with PVD2 patients. Moreover, PVD1 patients also exhibited higher anxiety and catastrophizing scores as demonstrated by questionnaires results. Further, neuroimaging differences between the two cohorts of patients were also confirmed. Gray matter (GM) density measured with neuroimaging differed between groups: Women with PVD1 showed significant GM density reduction in the left anterior cingulate cortex, parahippocampal gyrus, left inferior temporal gyrus, bilateral precuneus, right superior frontal gyrus, and some cerebellar areas. Women with PVD2 also showed GM density reduction, but of a minor magnitude and in fewer brain areas (the right superior occipital gyrus and the left inferior parietal cortex). In the same way, functional MRI scans documented more pronounced activation of pain-related areas (the postcentral gyrus, midcingulate cortex, insula, and thalamus) in PVD1 than PVD2 patients. The role of brain neuroimaging in CPP patients will be better elucidated in the next section (see Section 3). Another recent investigation further assessed neurophysiological modifications in a cohort of Provoked Vestibulodynia [126], as Pelvic Floor Muscle abnormalities were detected and matched with a surface Electromyographic evaluation. Pelvic Floor Muscles of the patients were stiff and more prone to increased resting and activated muscle tone (during stretching) than control subjects; all these abnormalities were documented by surface Electromyographic recording of anomalies. Recently, a new algometer for vestibular pain has been developed [127]; when compared with the Vulvalgesiometer for PPTs and Pain Pressure Tolerance (PPTol) outcomes, this new developed algometer was found to be a reliable and valid instrument for measuring PPTs and PPTols in the vestibular area in women with PVD, and therefore this technology may be promising for pinpointing treatment mechanisms and efficacy. Lastly, thermal algometry for CPP assessment has been proposed [128, 129] in men with CPP. Two series of fast trains of perineal and front thigh thermal painful stimuli through a small thermal electrode were administered to the patients, in association with a computerized VAS registration. A lower thermal pain threshold was detected, and the authors speculated that these results were related to central sensitization processes; this idea was also supported by some studies regarding pudendal somatosensory evoked potentials (SEP) recordings. SEP evaluation was also recently performed in Chronic Prostatitis/Chronic Pelvic Pain Syndrome (CP/CPPS) patients [130]. For SEP recording, electrical stimuli were applied with penile ring electrodes for dorsal penile nerve stimulation; N50 latencies were significantly shortened in the patient group compared to the healthy controls, thus demonstrating a pathogenetic neural mechanism related to central sensitization processes underlying CP/CPPS. CP/CPPS patients were also examined by investigating both SEP and Electromyography (EMG) [131]. Intramuscular EMG, right and left bulbocavernosus reflex, and cortical SEP during stimulation of pudendal nerve were evaluated, and a high frequency of abnormal neurophysiological patterns in the absence of clinical neurological disease was found in these patients.

Some authors investigated CPP/CPPS patients with Neurometer Current Perception Threshold (CPT) analysis in order to objectivate neuropathic features in such patients. Neurometer evaluations were firstly performed in male and female healthy subjects in order to assess Current Perception Thresholds in the lower urinary tract [132]. Neurometer CPT can quantify the functional integrity of myelinated (with both large or small diameter) and unmyelinated sensory fibers. Patients with Painful Bladder Syndrome/Interstitial Cystitis or overactive bladder have been evaluated [133–135] with stimuli delivered in the form of alternating currents with different stimulation frequency in order to evaluate Aß, A*δ*, and C fibers and producing threshold values for the current perception in such bladder afferent fibers. Men suffering from CPPS were also evaluated using Neurometer CPT [136] although no significative differences were found between CPPS patients and matched healthy controls.

Unfortunately, only a limited number of investigations have been performed in order to reveal neurophysiological abnormalities in CPP/CPPS patients. Laser Evoked Potentials (LEPs) are one of the most reliable laboratory tools to objectivate and diagnose neuropathic pain [137], but no studies investigating CPP/CPPS have been performed so far.

*Take-Home Message*. QST provides integration for the regular neurological examination, giving the opportunity to better objectivate neurophysiological abnormalities in CPP/CPPS patients by establishing pain thresholds. Algometry allows optimal standardization of QST, as it gives reliable and precise quantification of the administered stimulus and the subsequent threshold. Electrophysiological evaluation, with SEP and/or EMG, may further help to objectivate neurophysiological anomalies in CPP/CPPS patients. Despite these promising features, only a few studies have been performed, and therefore further investigations are warranted to elucidate the real importance of these tools when evaluating CPP/CPPS patients.

## 3. New Perspectives: The Role of Brain Neuroimaging

In recent years, Magnetic Resonance Imaging (MRI) and functional MRI (fMRI) have been suggested as tools to diagnose and objectivate chronic pain, as characteristic abnormalities have been detected in several chronic pain syndromes [138–141].

In the same manner, several studies assessed brain abnormalities with fMRI protocols in different CPP/CPPS patients both for pathophysiological and for therapeutic meanings. In particular, Endometriosis patients were evaluated with different neuroimaging protocols. Proton spectroscopy and seed-based resting functional connectivity MRI were used to determine whether women with Endometriosis display differences in insula excitatory neurotransmitter concentrations or intrinsic brain connectivity to other pain-related brain regions [142]. Compared to age-matched pain-free controls, women with Endometriosis-associated CPP displayed increased levels of combined glutamine-glutamate (Glx) within the anterior insula and greater anterior insula connectivity to the medial prefrontal cortex (mPFC). Increased connectivity between these regions was positively correlated with anterior insula Glx concentrations and also with psychopathological outcomes. The same group previously investigated changes in regional Gray Matter (GM) volume in women with Endometriosis-related CPP [143] with voxel-based morphometry, which is a neuroimaging tool that allows brain volumetric comparison between two groups of patients. Different groups of patients with CPP and/or Endometriosis were compared with healthy controls. Women with Endometriosis-associated CPP displayed decreased GM volume in several brain regions when compared with healthy women: the left thalamus, left cingulate gyrus, right putamen, and right insula showed significant volume loss. The areas discovered to be relatively “atrophic” are involved in pain processing, and pain cognitive/emotional cortical pathways (the so-called “pain matrix”) are often affected in CPP patients as shown by several structural and functional neuroimaging investigations. Women with CPP without Endometriosis were also evaluated and indeed showed GM volume loss in the left thalamus when compared with the healthy controls group. Notably, women with Endometriosis without CPP symptoms showed no volumetric differences in comparison with healthy women. The authors therefore speculated that different physiopathological mechanisms underlie the different MRI findings, as CPP alone (in women without Endometriosis) appeared to be responsible for volumetric GM decrease although of less magnitude than CPP accompanied by Endometriosis.

Furthermore, brain connectivity was evaluated in Endometriosis patients with resting-state fMRI imaging, before and after psychotherapeutic protocols [144]. It was identified as a cortical network comprising the right anterolateral hippocampus (a region modulating the hypothalamic-pituitary-adrenal axis and somatosensory, viscerosensory, and interoceptive brain regions), and a reduction in connectivity predicted therapy-induced improvement in patients' anxiety.

Besides patients with Endometriosis, patients with urologic CPP were evaluated in several neuroimaging protocols. The Multidisciplinary Approach to the Study of Chronic Pelvic Pain (MAPP) Research Network (http://www.mappnetwork.org/) is a multicenter collaborative research group established to conduct integrated studies on participants with urologic Chronic Pelvic Pain Syndrome (UCPPS) [145]. The aim of the group was to evaluate etiology; natural history; and clinical, demographic, and behavioural characteristics of urological CPP patients. A branch of the MAPP Network compared resting-state brain activity analysis, which is a functional neuroimaging tool that provides estimation of brain functional connectivity (FC), between women with urological CPPS and healthy women. Several FC abnormalities were observed in the CPPS cohort. First of all, significantly decreased FC was reported in 2 regions of the posterior medial cortex (the posterior cingulate cortex and the left precuneus) [146]. The left precuneus also showed decreased FC to several prefrontal and parietal cortical regions involved in cognitive tasks, executive functioning, set-shifting processes, and reward processes. Furthermore, increased FC between the posterior cingulate cortex and insular cortex, dorsolateral prefrontal cortex, thalamus, globus pallidus, putamen, amygdala, and hippocampus was detected in the CPPS group. Notably, these involved areas are part of a cortical network that processes cognitive and emotional characteristics of several kinds of sensory information, including pain. These results show how CPPS affects brain connectivity in different regions and pathways and provide new insights into pathophysiological CPP/CPPS mechanisms. Another branch of the MAPP study compared UCPPS patients with healthy controls and Irritable Bowel Syndrome (IBS) patients [147, 148]. White Matter differences were examined, with specific neuroimaging tools developed to investigate axonal and White Matter abnormalities such as Fractional Anisotropy (FA), Generalized Anisotropy (GA), Mean Diffusivity (MD), and Track Density. All the investigations documented remarkable differences between CPPS patients and healthy controls. Reduced FA was reported in the genu and splenium of the corpus callosum of UCPPS patients compared with healthy women. Conversely, increased FA was documented in some thalamic and basal ganglia regions, as well as in a tract included in the left somatosensory cortex, which may be associated with somatosensory integration of pelvic stimuli. MD and GA also were altered in CPPS patients, as higher MD in the basal ganglia, right frontal lobe, bilateral corona radiata fibers, and genu of the corpus callosum was detected in CPPS women when compared to controls; moreover, GA analysis showed decreased GA in several brain regions, mostly in corpus callosum and corona radiata [147]. Similar results were obtained by another MAPP group of investigators, as reduced FA was observed in several regions involved in pain perception, processing, modulation, and emotional integration such as right corticospinal tract and right anterior thalamic radiation [148]. Both research groups moreover distinguished these abnormalities from the IBS group MRI findings, as neuroimaging findings appear to be more pronounced and widespread in UCPPS patients when compared with IBS patients. Thus, the authors speculated that different pain syndromes may elicit different changes in brain structure and functioning.

The MAPP study group also enrolled men with Chronic Prostatitis/Chronic Pelvic Pain Syndrome comparing them to healthy controls. With resting-state FC analysis, significant group difference was observed in the functional connectivity between pelvic-motor and the right posterior insula [149]. It was therefore speculated that brain networks controlling Pelvic Floor Muscles reflected altered resting Pelvic Floor Muscle activity in men with CP/CPPS compared to healthy controls. Another recent functional neuroimaging investigation [150] revealed alterations in Regional Homogeneity of resting-state cerebral activity in patients with Chronic Prostatitis/Chronic Pelvic Pain Syndrome. Regional Homogeneity is a voxel-based measure of brain activity which evaluates the similarity or synchronization between a voxel (of a given brain region) and its nearest neighbors, based on the hypothesis that intrinsic brain activity is manifested by clusters of voxels rather than single voxels. It was found that CP/CPPS patients had significantly decreased Regional Homogeneity in the bilateral anterior cingulate cortices, insulae, and right medial prefrontal cortex; on the other hand, significantly increased Regional Homogeneity in the brainstem and right thalamus was detected by comparing CP/CPPS values with those of healthy controls. All these structures are thought to be relevant to pain processing and modulation. Notably, left anterior cingulate cortex, bilateral insular cortices, and brainstem abnormalities also correlated with pain symptoms and pain scale scores. CP/CPPS patients were also evaluated with another functional neuroimaging technique, Arterial Spin Labelling, which allows investigating slow varying changes in brain function [151]. Therapy-related longitudinal modifications in Arterial Spin Labelling were assessed after a cycle of sono-electromagnetic therapy or placebo. It was found that responders and nonresponders to the therapeutic protocol showed different Cerebral Blood Flow (CBF) modifications as documented by Arterial Spin Labelling. In detail, nonresponder patients showed CBF upregulation in the hippocampus, whereas responders underscored CBF downregulation in the prefrontal cortex and anterior cingulate cortex and upregulation in the dorsolateral prefrontal cortex. Responders therefore, according to the positive therapeutic outcome, showed a modulation in CBF in regions involved in pain processing and modulation. Moreover, structural brain modifications have also been investigated in CP/CPPS patients [152, 153], and a significative reduction of relative Gray Matter volume in the anterior cingulate cortex of the dominant hemisphere, compared to healthy controls, was documented [152]. Also, density alterations of Gray Matter in pain relevant regions (anterior insula and anterior cingulate cortices) were evident in a different cohort of CP/CPPS patients [153]; the findings positively correlated with pain intensity and duration of chronic pain.

Vulvodynia patients have also been recently investigated with fMRI scanning (resting-state analysis) [154], and alterations in resting-state were compared with those of HCs and a chronic pain control group (IBS). Intrinsic connectivity of regions comprising sensorimotor, salience, and default mode resting-state networks was examined, and subjects with Vulvodynia showed substantial alterations in the intrinsic connectivity of these networks compared with both healthy controls and Irritable Bowel Syndrome patients. The intrinsic connectivity of many of the regions showing group differences during rest was moderately associated with clinical symptom reports in CPP patients.

Primary Dysmenorrhea (PDM) women were also enrolled in several neuroimaging protocols. Voxel-based morphometry analysis showed abnormal volumetric decreases in regions involved in pain transmission and higher level sensory processing and affected regulation, while volumetric increases were found in regions involved in pain modulation and in regulation of endocrine function [155]. The same authors also investigated PDM patients using fluorodeoxyglucose positron emission tomography [156]. Results showed increased activity in prefrontal/orbitofrontal regions and left ventral posterior thalamus, while decreased activity was mainly observed in sensorimotor regions of the left hemisphere at onset compared to offset of PDM. These results were specific to menstrual pain and were not found in menstrual matched controls. Orbitofrontal activities were positively related to subjective pain ratings, while somatosensory activities were negatively related to these ratings. Another recent study investigated PDM patients with FC analysis; also, the analysis of Single Nucleotide Polymorphism (SNP) of mu-opioid receptor was performed and matched with fMRI results [157]. It was found that G allele SNP carriers, in comparison to AA homozygotes, exhibited functional hypoconnectivity between the anterior cingulate cortex (ACC) and Periaqueductal Gray (PAG). Furthermore, G allele carriers lost the correlation with spontaneous pain experience and exhibited dysfunctional Descending Pain Modulating System as confirmed by PAG-seeded FC dynamics. Similar mismatches with the Descending Pain Modulating System were already previously detected in Brain-Derived Neurotrophic Factor (BDNF) Val66Met SNP carriers by the same authors [158] using resting-state FC examinations.

All these investigations document how CPP and CPPS, rather than being merely urogynaecological syndromes, have fundamental neural pathophysiological mechanisms, as neuroimaging abnormalities have been identified in several cohorts of patients. These alterations may possibly reflect sensitization processes and neuroplastic modifications in Central Nervous System, which are subsequent to the chronic painful stimulation arising from the periphery. It is also possible that some patients, that is, SNP carriers, have alterations in cortical circuitry that predispose them to the development of chronic pain syndromes such as CPP. Of course, other researches are warranted to better understand the alterations in CPP patients' brain and to properly match the neuroradiological findings with clinical and possibly genetic assessments. Nevertheless, brain neuroimaging in CPP patients appears to be a promising field as it offers the future possibility of a reliable noninvasive marker of CPP and CPP severity.

*Take-Home Message*. Being similar to other patients with chronic pain conditions, CPP/CPPS patients showed several structural and functional brain neuroimaging abnormalities. Anomalies were found in both male and female CPP/CPPS patients. Therefore, brain neuroimaging appears to be a promising tool for further evaluation of CPP/CPPS patients both for pathophysiological and for diagnostic/assessment purposes, although further researches are surely warranted to fully understand the correlations between CPP/CPPS symptoms and brain functional and structural anomalies.

## 4. Conclusions

Despite the development of several screening and diagnostic tools, pain evaluation and objectivation still remain not seldom troublesome in CPP/CPPS. Nevertheless, a full and correct assessment of patients' painful experience can help physicians to properly develop appropriate therapeutic protocols specially tailored for those patients, taking into account pain symptoms as well as eventual comorbidities, psychological features, and functional QoL status. Future investigations are warranted to ameliorate diagnostic tools accuracy and to correlate instrumental findings.
